# The Incidence of Senile Cataract and Glaucoma is Increased in Patients with Plasma Cell Dyscrasias: Etiologic Implications

**DOI:** 10.1038/srep28500

**Published:** 2016-06-22

**Authors:** Kari Hemminki, Asta Försti, Raimo Tuuminen, Otto Hemminki, Hartmut Goldschmidt, Kristina Sundquist, Jan Sundquist, Xinjun Li

**Affiliations:** 1 Division of Molecular Genetic Epidemiology, German Cancer Research Center (DKFZ), Im Neuenheimer Feld 580, D-69120, Heidelberg, Germany; 2 Center for Primary Health Care Research, Lund University, 205 02 Malmö, Sweden; 3 Department of Ophthalmology, Kymenlaakso Central Hospital, Kotka, Finland; 4 Department of Urology, Kymenlaakso Central Hospital, Kotka, Finland; 5 Cancer Gene Therapy Group, Department of Pathology and Transplantation Laboratory and Haartman Institute, University of Helsinki, Finland; 6 Department of Internal Medicine V, University of Heidelberg, Germany; 7 National Centre for Tumor Diseases, Heidelberg, Germany; 8 Stanford Prevention Research Center, Stanford University School of Medicine, Stanford, California 94305-5705, USA

## Abstract

Plasma cell dyscrasias, including monoclonal gammopathy of undetermined significance (MGUS), multiple myeloma (MM), Waldenström macroglobulinemia (WM) and light chain AL amyloidosis, are characterized by clonal expansion of plasma cells which produce a vast amount of an immunoglobulin-derived M-protein. We noted that MGUS diagnosis often coincided with diagnoses of senile cataract and glaucoma and tested the associations of MGUS, MM, WM and AL amyloidosis with subsequent eye diseases identified from the Swedish patient registers between 1997 and 2012. Standardized incidence ratios (SIRs) for senile cataract was significantly increased to 1.80 after MGUS, 1.70 after MM, 1.85 after WM and 2.31 after AL amyloidosis. The SIR for glaucoma was 1.60 after MGUS, 1.76 after WM and 2.18 after AL amyloidosis. All SIRs decreased systematically from age below 60 years to over 79 years, but most risks were also significant in age group over 79 years. The M-protein and the related increase in blood viscosity could be a novel etiologic discovery for these common eye diseases. As MGUS prevalence is around 3% at 60 years and close to 10% at age over 80 years, its contribution to the eye disease burden is expected to be remarkably high.

Senile cataract and glaucoma are common diseases, the incidence of which increases steeply past the age of 60 years^1^,^2^. Both of the diseases are the major causes of blindness worldwide, cataract being the leading one^3^. Among Swedes older than 39 years of age, 9.0% were diagnosed with cataract and 2.8% with glaucoma during a period of 11 years^4^. Senile cataract is defined as the opacity of the lens of the eye or its capsule, with no obvious cause, occurring in people over the age of 50 years^5^. The opacity is due to protein aggregates that are formed for unknown reasons. Protein aggregates may be located in various parts of the lens structure, which constitute the types of cataract: nuclear, cortical and posterior subcapsular^6^. The nuclear type is the most common one but in many patients mixed types are found^1^. There are some risk factors that facilitate the age dependent opacification, including family history, diabetes, smoking and solar exposure^1^,^6^. The composition of the lens opacity is not known but structural modifications of crystallins (the proteins maintaining the transparency of the lens) are suspected to be involved and catalyzed by oxidative stress^7^,^8^. Glaucoma is a type of optic neuropathy characterized by indented appearance of the optic nerve, referred to as optic nerve cupping^9^. The cupping is a sign of damage to the optic nerve, which is often related to increased intraocular pressure. The cause of the elevated pressure may result from obstruction of the humoral drainage through the trabecular meshwork in the iridocorneal angle^5^. The main type of glaucoma is primary open-angle glaucoma in which the iridocorneal angle appears to be open as normal but the aqueous outflow may be obstructed; in the angle-closure glaucoma, however the drainage is usually blocked by the iris^10^.

We have had an interest, prior to commencing this study, in genetics and epidemiology of plasma cell dyscrasias, including multiple myeloma (MM), monoclonal gammopathy of undetermined significance (MGUS) and light chain AL amyloidosis^11^,^12^,^13^. MGUS is a pre-malignant precursor condition of MM, AL amyloidosis and, additionally, of Waldenström macroglobulinemia (WM) with a combined progression rare of 1% per year^14^,^15^,^16^. The prevalence of MGUS increases almost linearly with age (after 50 years) reaching 9.9% in the age group 80 to 89 years^17^. As MGUS is an asymptomatic condition it is diagnosed, often fortuitously, during a medical check-up for another cause, through serum paraprotein (M-protein) produced by a plasma cell clone^14^,^18^. We recently identified 11,233 MGUS patients from Swedish nationwide hospital records. Whilst in checking for other diagnoses, rendered synchronously with MGUS, we found that senile cataract and glaucoma were surprisingly more common than expected. We present here the detailed incidence relationship with plasma cell dyscrasia and these eye diseases, and suggest that they may open a novel etiologic window into mechanisms of senile cataract and glaucoma.

## Results

The numbers of people with the diagnosis of interest found in the Swedish medical registers are shown in Table 1. They range from 736,789 for senile cataract to 1530 for WM. Men outnumbered women for plasma cell dyscrasias, while the opposite was true for the eye diseases.

AL amyloidosis was diagnosed earlier (65 years) than the other conditions (72 to 76 years). The Outpatient Register was the main source of data for non-malignant conditions. Important for the uniformity of the follow-up period that 97% of senile cataract and 93% glaucoma data were obtained from the Outpatient Register covering years 2001 to 2012.

The suspected excess of senile cataract and glaucoma in plasma cell dyscrasia patients was entirely due to eye diseases diagnosed after the diagnoses of plasma cell dyscrasias. Thus all the following results refer to subsequent eye diseases.

In Table 2 subsequent risks of senile cataract are shown in plasma cell dyscrasia patients by sex, age at diagnosis of plasma cell dyscrasias and diagnosis defined by the third digit of the ICD-10 code. For all senile cataract the SIRs were between 1.70 and 1.85 after MGUS, MM and WM but higher (2.31) after AL amyloidosis. Male SIRs tend to be higher than female SIRs but none of the differences were significant (i.e., 95%CIs overlapped). All SIRs decreased systematically from age below 60 years to over 79 years. However, the decrease was less steep for MGUS than for the other plasma cell dyscrasias; the SIR was 7.30 in AL amyloidosis patients diagnosed at age below 60 years. Notably, all significant overall risks were also significant in age group over 79 years. Senile cataract diagnoses were further refined by the third digit of the ICD-10 code. Although many of the diagnoses were non-specific, senile nuclear cataract was increased after all plasma cell dyscrasias (after WM and AL amyloidosis of borderline significance, i.e., the lower 95%CI was close to 1.00). An important support to the diagnostic accuracy was that among senile cataract patients 69% had at least two visits with the same diagnosis. No reliable data on eye operations were available.

In Table 3 subsequent risks of glaucoma are shown in plasma cell dyscrasias by sex, age at diagnosis of plasma cell dyscrasias and diagnosis defined by the third digit of the ICD-10 code. The SIRs were significantly increased after all plasma cell dyscrasias, with the exception of MM patients. However, the SIRs for glaucoma were somewhat lower than those after senile cataract: 1.60 after MGUS, 1.76 after WM and 2.18 after AL amyloidosis. Yet the distributions by sex and diagnostic age were similar for the two eye diseases; the SIR for glaucoma was 7.64 in AL amyloidosis patients diagnosed at age below 60 years. Primary open-angle glaucoma was increased in both MGUS and AL amyloidosis patients. Some subcategories of glaucoma were vastly increased in AL amyloidosis patients, particularly for ‘glaucoma secondary to eye inflammation’ (N = 7, SIR 43.75, 95%CI 17.34–90.65). Among glaucoma patients, 73% had at least two visits with the same diagnosis and according to the data available from the Prescription Drug Register for years 2005 and 2012, 68% had received glaucoma medication.

**Figure 1:**
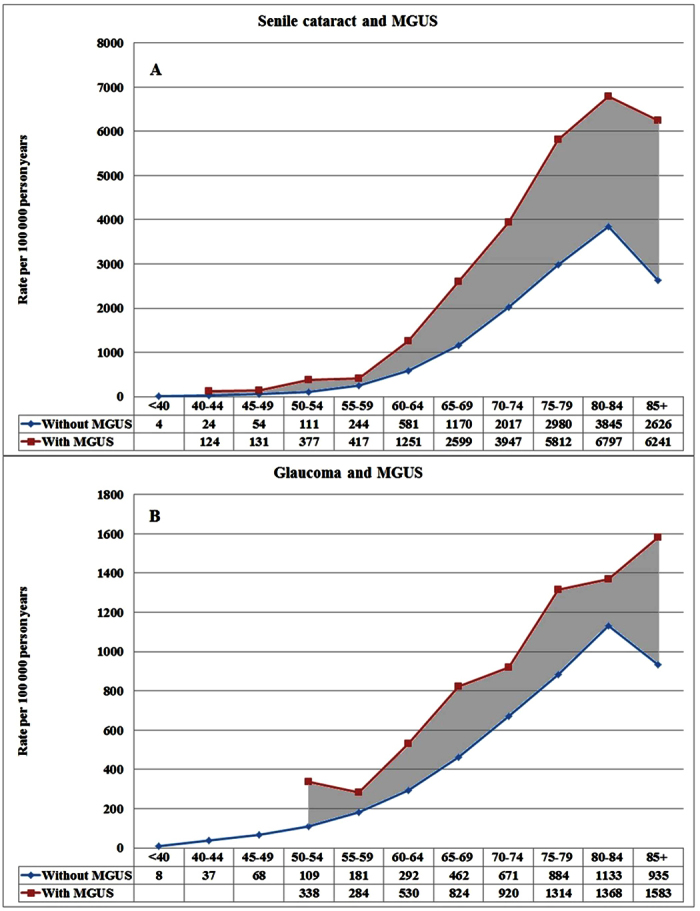
Incidence of subsequent senile cataract (**A**) and glaucoma (**B**) in MGUS patients compared to eye disease patients without prior MGUS.

Figure 1 shows the incidence of subsequent senile cataract and glaucoma in MGUS patients, compared to those without MGUS. For senile cataract (1711 with MGUS and 735,078 without MGUS) the incidence difference became apparent at age 40 years (Fig. 1A). At the maximum, at age 80 to 84 the incidence was almost doubled with MGUS background (6797 vs. 3845 per 100,000). For glaucoma (512 with MGUS and 298,910 without MGUS), a clear incidence difference was noted from age 50 onwards depending on the MGUS background (Fig. 1B). The incidence data on senile cataract and glaucoma after MM, WM and AL amyloidosis are shown in Supplementary Figures 1 to 3. For senile cataract the incidence rates was clearly higher after all plasma cell dyscrasias; for glaucoma the differences were smaller and for MM there was no difference. The highest recorded incidence was after AL amyloidosis, 7514 per 100,000 in senile cataract, and 2252 per 100,000 for glaucoma (Supplementary Figure 3). Some 15% of the combined number of senile cataract and glaucoma patients were diagnosed with both diseases in the current population.

In Supplementary Table 1, subsequent risk of senile cataract and glaucoma are shown by follow-up time from the diagnosis of plasma cell dyscrasia and age at diagnosis of the eye disease. The SIRs of senile cataract and glaucoma showed a U-shaped relationship to the length of the follow-up time. The highest risks were in the year following plasma cell dyscrasia diagnosis and 10 or more years after diagnosis. The SIRs were higher when senile cataract and glaucoma were diagnosed before the age of 60 years compared to a later diagnosis. For senile cataract or glaucoma following AL amyloidosis before age 60 years, the SIRs were 7.30 and 7.64, respectively.

## Discussion

The present results show convincing excess risk for subsequent senile cataract and glaucoma after four plasma cell dyscrasias, all of which are characterized by overproduction of immunoglobulin derived M-protein or light chains. In MGUS, the concentration is less than 3 g/dL and in MM it is above this limit. In WM, the M-protein is IgM at any increased concentration. In AL amyloidosis, the light chain is aggregated into amyloid particles. There are hardly any common risk factors, in addition to age, shared by plasma cell dyscrasias and the above mentioned eye diseases. A prolonged use of corticosteroids is a risk factor for senile cataract and glaucoma but no such medications are used in asymptomatic MGUS. Plasma cell dyscrasias show familial clustering but these are not known to encompass the studied eye diseases^19^. Thus the likely cause for the increased risk of senile cataract and glaucoma in plasma cell dyscrasia patients is the presence of the M-protein, which contributes to an increased viscosity of blood.

It has been estimated in the USA that an elevated level of M-protein is detectable on average more than 10 years before the diagnosis of MGUS^20^. As MGUS is a precursor condition for the other present plasma cell dyscrasias, the affected individuals may experience an increased M-protein concentration for some decades. High concentrations of M-protein increase the viscosity of blood, and plasma cell dyscrasias may be diagnosed as a hyperviscosity syndrome^21^. The type of M-protein influences viscosity and the large size of IgM helps to confer a higher intrinsic viscosity compared to IgG and IgA. This is the reason why hyperviscosity is more prevalent in WM, overexpressing only IgM, than in MM, overexpressing most commonly IgG. In the present study the risks were quite uniform following MGUS, MM or WM, which may be due to lower medical attention or less active notification of multiple medical conditions in patients with important primary disease (MM and WM). Eye diseases were most common in AL amyloidosis patients probably because amyloid aggregates would be expected to interfere with lens structures and eye fluid drainage systems. AL amyloidosis patients had a particularly high risk (43.75) for ‘glaucoma secondary to eye inflammation’, probably because of amyloids causing chronic irritation of the eye^22^,^23^.

A few personal risk factors of MGUS include black race, autoimmune disease, prior infections or exposure to herbicides^24^,^25^,^26^. MGUS is associated with the risk of bone fractures and of arterial and venal thrombosis^27^,^28^. A study in the Mayo clinic collected all medical diagnoses of 605 MGUS patients and compared these to local controls^29^. The associated diseases included plasma cell dyscrasias, superficial thrombophlebitis and bone diseases, such as vertebral and hip fractures as well as osteoporosis. In the appendix of that paper, the relative risk of senile cataract was given as 1.1 (N = 25) and that of primary open-angle glaucoma as 1.1 (N = 33)^29^. An imminent question is why the risk for these eye diseases was found significant only in the present study. One relevant point is that in the present study an increased risk for senile cataract and glaucoma was observed only following the four plasma cell dyscrasias, while in the cohort examined by Bida *et al*. the order of diagnoses is not clear^29^. Moreover, it is possible the catchment of senile cataracts was not complete in the study by Bida *et al*. because only 25 cases were found (4.1% of MGUS diagnoses), while in the present study the corresponding figure was 15.2% (1711/11233).

The lens of the eye is considered exquisitely sensitive to protein aggregation because the ambient protein concentration is the highest of any tissue and because the lens proteins are extremely long-lived^7^,^30^. Against this background it is somewhat surprising that conditions with increased blood viscosity or protein aggregation have not previously attracted formal studies in relation of cataract or glaucoma. However some case reports have appeared, including on ocular manifestations in MM with suggested immunoglobulin light chain deposition in ocular tissues^22^. Case reports of secondary glaucoma in MM patients have also been published and an author of a case report on a glaucoma patient with MGUS speculated that the patient might have developed a hypercoagulable syndrome and obstructed blood flow to the optic nerve^22^,^31^. Recurrent subconjuctival and periorbital bleeding was reported in AL amyloidosis^23^.

The present nation-wide study has the advantage of large patient numbers and statistical power. The data were collected from various sources with different starting times which may introduce some heterogeneity to the results. However, most importantly, the overwhelming proportion of eye disease data (~95%) was obtained from a uniform source, the Outpatient Register. The weakness of this source was that operation data were largely missing. The statistical method was based on indirect standardization which is particularly efficient when small numbers are analyzed^32^. In the present analysis various subgroups were small justifying the use of indirect standardization. Even though many overall results were relatively uniform with SIRs below 2.0, there were clear trends by sex, diagnostic age and subgroups. Importantly, SIRs were increased for subsequent but nor for prior eye diseases.

The present results provide unique clues to the pathophysiologic mechanisms of senile cataract and glaucoma, with indirect incrimination of high concentrations of M-protein facilitating lens opacification and obstruction of humoral drainage through the trabecular meshwork. In case of senile cataract, the results were homogeneous for the four plasma cell dyscrasia, with slightly higher risk after AL amyloidosis than the three others. The data were also consistent for glaucoma, except that MM was associated with no risk. MM, WM and AL amyloidosis are life-threatening diseases, and the excess eye diseases cause an additional illness burden for the patients. However, the findings on MGUS have main implications to the understanding of senile cataract and glaucoma etiology. As MGUS is underdiagnosed its contribution to the associated eye diseases is much larger than the results show at the first glance. With a true prevalence of 10%, by age 80 years, the population attributable fraction of MGUS in senile cataract and glaucoma would probably surpass any known causes. For a proper population level risk estimation, M-protein levels need to be measured in senile cataract and glaucoma patients.

The current recommendations for MGUS call for follow-up by serum electrophoresis, if the likelihood of progression to MM, WM or AL amyloidosis is high^14^. The risk stratification considers M-protein concentration and type^14^. Most of the diagnosed cases are low-risk individuals who would not be followed-up after a control electrophoresis has shown a stable M-protein concentration. It is unlikely that the risk of senile cataract and glaucoma would change the recommendations for follow-up. However, a number of other diseases have also been associated with MGUS, including bone diseases, neuropathy and thrombosis^27^,^28^,^29^. With all these health risks associated with MGUS an active follow-up would be justified if efficient and risk-free interventions were available. The paradigm in oncology is to remove or destroy the precursor lesion before the transformation to malignancy or metastatic spread has occurred. The imminent challenge in MGUS research is to find ways of selectively and safely targeting the still benign M-protein producing plasma cell clone.

## Patients and Methods

Persons diagnosed with MGUS, AL-amyloidosis, senile cataract and glaucoma were identified from the Swedish Hospital Discharge Register from 1997 through 2012, and the Outpatient Register from 2001 through 2012 (Table 1). Both registers are nationwide. Only the first diagnosis for each person of MGUS, AL amyloidosis, senile cataract or glaucoma in either register was included. MM and WM were obtained from the Swedish Cancer Registry from the period 1997 through 2012. The International Classification of Diseases (ICD) version 10 was used to identify MGUS (D47.2), MM (C90), WM (C88), senile cataract (H25) and glaucoma (H40). The standard diagnostic criteria for MGUS was the presence of M-protein in serum at less than 3 g/dL, fewer than 10% plasma cells in the bone marrow, no evidence of other lymphoproliferative disorders, and lack of symptoms in relation to the monoclonal gammopathy^33^. AL amyloidosis was defined by codes E85.4, E85.8 and E85.9. In addition, these patients had to be treated with melphalan, with data recorded in the Prescription Drug Register (in operation since 2005) because the diagnostic codes for AL amyloidosis were not specific as discussed^34^. Information from the registers was linked at the individual level using the national civic registration number, allowing practically a complete linkage to for the entire population. In the linked dataset, the number was replaced with a serial number to ensure anonymity.

Person-years were calculated from the start of follow-up on January 1^st^ 1997/2001 until diagnosis of the relevant disease, death, emigration, or the end of the study (December 31^st^, 2012) whichever came first. We used SAS version 9.3 for the statistical analyses. Standardized incidence ratios (SIRs) were calculated as the ratio of observed to expected number of cases, for the eye diseases between 1997 and 2012. The expected numbers were calculated for all individuals without a personal history of plasma cell dyscrasia. The rates were standardized by 5-year-age, gender, period (5-year-groups), socioeconomic status and residential area. The covariates were selected based on their known or assumed influence on the incidence of the diseases under study. Before adopting the full model they were individually added to the basic model which considered age and period only; however none of the added covariates essentially changed the risk estimates. The 95% confidence interval (95%CI) of the SIR was calculated assuming a Poisson distribution. The results are called statistically ‘significant’ when the 95%CIs do not reach an SIR of 1.00.

### Ethical statement

The study was approved by the Ethical Committee of Lund University and the study was conducted in accordance with the approved guidelines.

## Additional Information

**How to cite this article**: Hemminki, K. *et al*. The Incidence of Senile Cataract and Glaucoma is Increased in Patients with Plasma Cell Dyscrasias: Etiologic Implications. *Sci. Rep.* **6**, 28500; doi: 10.1038/srep28500 (2016).

## Supplementary Material

Supplementary Information

## Figures and Tables

**Table 1:** Number of cases, sex, median diagnosis age and origin of data for plasma cell dyscrasia and eye disease patients.

	MGUS	MM	>Waldenström	AL amyloidosis	Senile cataract	Glaucoma
Total	11,233	9666	1530	3538	736,789	299,122
Gender, males (%)	5766 (51%)	5300 (55%)	907 (59%)	1899 (54%)	283,883 (39%)	122,371 (41%)
Median diagnosis age	72	72	74	65	76	74
Data origin						
Cancer Registry		9666	1530			
Hospital Diagnosis Register	1895			2204	18,454	19,730
Outpatient Register (%)	9338 (83%)			1334 (38%)	718,335 (97%)	279,392 (93%)

MGUS: Monoclonal gammopathy of undetermined significance; MM: Multiple myeloma.

**Table 2:** Subsequent risks of senile cataract in MGUS, MM, Waldenström, and AL amyloidosis patients.

	MGUS				MM				Waldenström				AL amyloidosis			
	O	SIR	95% CI		O	SIR	95% CI		O	SIR	95% CI		O	SIR	95% CI	
**All**	1711	**1.80**	**1.71**	**1.88**	891	**1.70**	**1.59**	**1.81**	237	**1.85**	**1.63**	**2.11**	338	**2.31**	**2.07**	**2.57**
Gender																
Men	771	**1.80**	**1.67**	**1.93**	439	**1.80**	**1.64**	**1.98**	132	**1.91**	**1.60**	**2.27**	161	**2.50**	**2.13**	**2.92**
Women	940	**1.80**	**1.68**	**1.91**	452	**1.61**	**1.46**	**1.76**	105	**1.78**	**1.46**	**2.16**	177	**2.16**	**1.85**	**2.50**
Age at diagnosis (years)																
<60	32	**2.04**	**1.40**	**2.89**	30	**3.17**	**2.14**	**4.53**	5	**3.31**	**1.04**	**7.79**	40	**7.30**	**5.21**	**9.95**
60–79	967	**1.88**	**1.76**	**2.00**	536	**1.77**	**1.63**	**1.93**	137	**2.10**	**1.77**	**2.49**	215	**2.29**	**1.99**	**2.62**
80+	712	**1.68**	**1.56**	**1.81**	325	**1.53**	**1.37**	**1.71**	95	**1.55**	**1.26**	**1.90**	83	**1.77**	**1.41**	**2.19**
Subtype																
Senile incipient cataract (H25.0)	282	**1.80**	**1.59**	**2.02**	151	**1.64**	**1.39**	**1.93**	43	**1.93**	**1.40**	**2.60**	61	**2.59**	**1.98**	**3.33**
Senile nuclear cataract (H25.1)	111	**1.75**	**1.44**	**2.11**	53	**1.45**	**1.08**	**1.89**	16	1.70	0.97	2.76	15	1.70	0.95	2.81
Other senile cataract (H25.2, H25.8)	28	**1.88**	**1.25**	**2.71**	20	**2.11**	**1.29**	**3.26**	3	1.36	0.26	4.02	10	**4.20**	**2.00**	**7.76**
Senile cataract, unspecified (H25.9)	1290	**1.80**	**1.70**	**1.90**	667	**1.73**	**1.60**	**1.86**	175	**1.86**	**1.60**	**2.16**	252	**2.26**	**1.99**	**2.56**

MGUS: Monoclonal gammopathy of undetermined significance; MM: Multiple myeloma.

O = Observed cases; SIR = Standardized incidence ratio; CI = Confidence intervals.

Bold type: 95% confidence interval does not include 1.00.

**Table 3:** Subsequent risks of glaucoma in MGUS, MM, Waldenström, and AL amyloidosis patients.

	MGUS				MM				Waldenström				AL amyloidosis			
	O	SIR	95% CI		O	SIR	95% CI		O	SIR	95% CI		O	SIR	95% CI	
**All**	512	**1.60**	**1.47**	**1.75**	207	1.11	0.97	1.28	81	**1.76**	**1.40**	**2.19**	118	**2.18**	**1.81**	**2.62**
Gender																
Men	227	**1.59**	**1.39**	**1.81**	100	1.13	0.92	1.38	48	**1.94**	**1.43**	**2.57**	62	**2.49**	**1.91**	**3.19**
Women	285	**1.61**	**1.43**	**1.81**	107	1.10	0.90	1.33	33	**1.55**	**1.07**	**2.18**	56	**1.92**	**1.45**	**2.50**
Age at diagnosis (years)																
<60	22	**1.80**	**1.13**	**2.73**	12	1.54	0.79	2.69	4	3.20	0.83	8.27	36	**7.64**	**5.35**	**10.59**
60–79	283	**1.66**	**1.47**	**1.86**	120	1.13	0.93	1.35	41	**1.76**	**1.26**	**2.39**	55	**1.60**	**1.20**	**2.08**
80+	207	**1.52**	**1.32**	**1.74**	75	1.05	0.82	1.31	36	**1.67**	**1.17**	**2.31**	27	**1.82**	**1.20**	**2.65**
Subtype																
Glaucoma suspect (H40.0)	248	**1.78**	**1.56**	**2.01**	83	1.09	0.87	1.36	38	**2.12**	**1.50**	**2.91**	40	**1.63**	**1.16**	**2.22**
Primary open-angle glaucoma (H40.1)	182	**1.35**	**1.16**	**1.56**	84	1.01	0.81	1.26	29	1.38	0.93	1.99	45	**2.01**	**1.46**	**2.69**
Primary angle-closure glaucoma (H40.2)	8	1.85	0.79	3.66	2	0.73	0.07	2.69	1	1.59	0.00	9.10	1	1.27	0.00	7.26
Glaucoma secondary to other eye disorders (H40.5)	16	**2.25**	**1.28**	**3.66**	9	2.12	0.96	4.04	2	1.80	0.17	6.63	10	**8.47**	**4.04**	**15.64**
Other glaucoma (H40.3, H40.4, H40.6, and H40.8)	4	2.45	0.64	6.35	1	0.95	0.00	5.46	0				9	**26.47**	**12.00**	**50.47**
Glaucoma, unspecified (H40.9)	54	**1.66**	**1.25**	**2.17**	28	1.46	0.97	2.11	11	**2.10**	**1.04**	**3.78**	13	**2.74**	**1.45**	**4.70**

MGUS: Monoclonal gammopathy of undetermined significance; MM: Multiple myeloma.

O = Observed cases; SIR = Standardized incidence ratio; CI = Confidence intervals.

Bold type: 95% confidence interval does not include 1.00.
