# Supplementary material for: The Incidence of Senile Cataract and Glaucoma is Increased in Patients with Plasma Cell Dyscrasias: Etiologic Implications
Source: Sci Rep. 2016 Jun 22;6:28500. doi: 10.1038/srep28500 (PMC4916420; doi:10.1038/srep28500)

**Supplementary Material**

**THE INCIDENCE OF SENILE CATARACT AND GLAUCOMA IS INCREASED IN PATIENTS WITH PLASMA CELL DYSCRASIAS: ETIOLOGIC IMPLICATIONS**

**Authors**

Kari Hemminki, Asta Försti, Raimo Tuuminen, Otto Hemminki, Hartmut Goldschmidt Kristina Sundquist, Jan Sundquist and Xinjun Li

**Contents**

Result, p. 2

Supplementary Table 1, p.3: Subsequent risks of senile cataract (ICD-10 H25) and glaucoma (ICD-10 H40) in MGUS, MM, Waldenström, and AL amyloidosis patients according to follow-up time.

Supplementary Figure 1, p. 4: Incidence of subsequent senile cataract (A) and glaucoma (B) in myeloma patients compared to eye disease patients without subsequent myeloma.

Supplementary Figure 2, p. 5: Incidence of subsequent senile cataract (A) and glaucoma (B) in Waldenström patients compared to eye disease patients without subsequent Waldenström.

Supplementary Figure 3, p. 6: Incidence of subsequent senile cataract (A) and glaucoma (B) in AL amyloidosis patients compared to eye disease patients without subsequent AL amyloidosis.

RESULTS

In Supplementary Table 1, subsequent risk of senile cataract and glaucoma are shown by follow-up time from the diagnosis of plasma cell dyscrasia. The SIRs of senile cataract and glaucoma showed a U-shaped relationship to the length of the follow-up time. The highest risks were in the year following plasma cell dyscrasia diagnosis and 10 or more years after diagnosis.

The incidence data on senile cataract and glaucoma after MM, WM and AL amyloidosis are shown in Supplementary Figures 1 to 3. For senile cataract the incidence rates was clearly higher after all plasma cell dyscrasias; for glaucoma the differences were smaller and for MM there was no difference. The highest recorded incidence was after AL amyloidosis, 7514 per 100,000 in senile cataract, and 2252 per 100,000 for glaucoma (Supplementary Figure 3).

| **Supplementary Table 1. Subsequent risks of senile cataract (ICD-10 H25) and glaucoma (ICD-10 H40) in MGUS, MM, Waldenström, and AL amyloidosis patients according to follow-up time** | | | | | | | | | | | | | | | | | | | | |  | | | |
| --- | --- | --- | --- | --- | --- | --- | --- | --- | --- | --- | --- | --- | --- | --- | --- | --- | --- | --- | --- | --- | --- | --- | --- | --- |
|  | MGUS | | | |  | MM | | | |  | Waldenström | | | |  | AL amyloidosis | | | |  | |  |  |  |
|  | O | SIR | 95% CI | |  | O | SIR | 95% CI | |  | O | SIR | 95% CI | |  | O | SIR | 95% CI | |  | |  |  |  |
| **Senile cataract (H25)** |  |  |  |  |  |  |  |  |  |  |  |  |  |  |  |  |  |  |  |  | |  |  |  |
| All | 1711 | **1.80** | **1.71** | **1.88** |  | 891 | **1.70** | **1.59** | **1.81** |  | 237 | **1.85** | **1.63** | **2.11** |  | 338 | **2.31** | **2.07** | **2.57** |  | |  |  |  |
| Follow-up time |  |  |  |  |  |  |  |  |  |  |  |  |  |  |  |  |  |  |  |  | |  |  |  |
| < 1 year | 340 | **3.31** | **2.97** | **3.68** |  | 192 | **2.24** | **1.93** | **2.58** |  | 36 | **2.67** | **1.87** | **3.70** |  | 68 | **3.38** | **2.63** | **4.29** |  | |  |  |  |
| 1-2 years | 546 | **1.66** | **1.52** | **1.80** |  | 286 | **1.38** | **1.22** | **1.54** |  | 71 | **1.78** | **1.39** | **2.25** |  | 102 | **2.14** | **1.75** | **2.60** |  | |  |  |  |
| 3-9 years | 762 | **1.55** | **1.44** | **1.67** |  | 385 | **1.80** | **1.62** | **1.99** |  | 114 | **1.68** | **1.39** | **2.02** |  | 144 | **2.05** | **1.73** | **2.42** |  | |  |  |  |
| ≥ 10 years | 63 | **2.13** | **1.64** | **2.73** |  | 28 | **1.73** | **1.15** | **2.50** |  | 16 | **2.37** | **1.35** | **3.86** |  | 24 | **2.84** | **1.82** | **4.23** |  | |  |  |  |
| **Glaucoma (H40)** |  |  |  |  |  |  |  |  |  |  |  |  |  |  |  |  |  |  |  |  | |  |  |  |
| All | 512 | **1.60** | **1.47** | **1.75** |  | 207 | 1.11 | 0.97 | 1.28 |  | 81 | **1.76** | **1.40** | **2.19** |  | 118 | **2.18** | **1.81** | **2.62** |  | |  |  |  |
| Follow-up time |  |  |  |  |  |  |  |  |  |  |  |  |  |  |  |  |  |  |  |  | |  |  |  |
| < 1 year | 119 | **3.33** | **2.76** | **3.98** |  | 47 | **1.50** | **1.10** | **2.00** |  | 15 | **2.95** | **1.64** | **4.87** |  | 29 | **3.69** | **2.47** | **5.30** |  | |  |  |  |
| 1-2 years | 159 | **1.41** | **1.20** | **1.65** |  | 66 | 0.88 | 0.68 | 1.12 |  | 26 | **1.73** | **1.13** | **2.54** |  | 25 | 1.35 | 0.87 | 2.00 |  | |  |  |  |
| 3-9 years | 207 | **1.28** | **1.11** | **1.47** |  | 87 | 1.17 | 0.93 | 1.44 |  | 37 | **1.55** | **1.09** | **2.14** |  | 55 | **2.18** | **1.64** | **2.84** |  | |  |  |  |
| ≥ 10 years | 27 | **2.85** | **1.88** | **4.16** |  | 7 | 1.34 | 0.53 | 2.77 |  | 3 | 1.40 | 0.26 | 4.13 |  | 9 | **3.61** | **1.64** | **6.89** |  | |  |  |  |
|  |  |  |  |  |  |  |  |  |  |  |  |  |  |  |  |  |  |  |  |  | |  |  |  |
| MGUS: Monoclonal gammopathy of undetermined significance; MM: Multiple myeloma.  O = Observed cases; SIR = Standardized incidence ratio; CI = Confidence intervals. | | | | | | | | | | | |  |  |  |  |  |  |  |  |  | |  |  |  |
| Bold type: 95% confidence interval does not include 1.00. | | | | | | | | | | | | | | | | | | | |  | |  |  |  |

**Supplementary Figure legends:**

**Supplementary Figure 1. Incidence of subsequent senile cataract (A) and glaucoma (B) in myeloma patients compared to eye disease patients without subsequent myeloma**

**Supplementary Figure 2. Incidence of subsequent senile cataract (A) and glaucoma (B) in Waldenström patients compared to eye disease patients without subsequent Waldenström.**

**Supplementary Figure 3. Incidence of subsequent senile cataract (A) and glaucoma (B) in AL amyloidosis patients compared to eye disease patients without subsequent AL amyloidosis.**

Supplementary Fig 1.


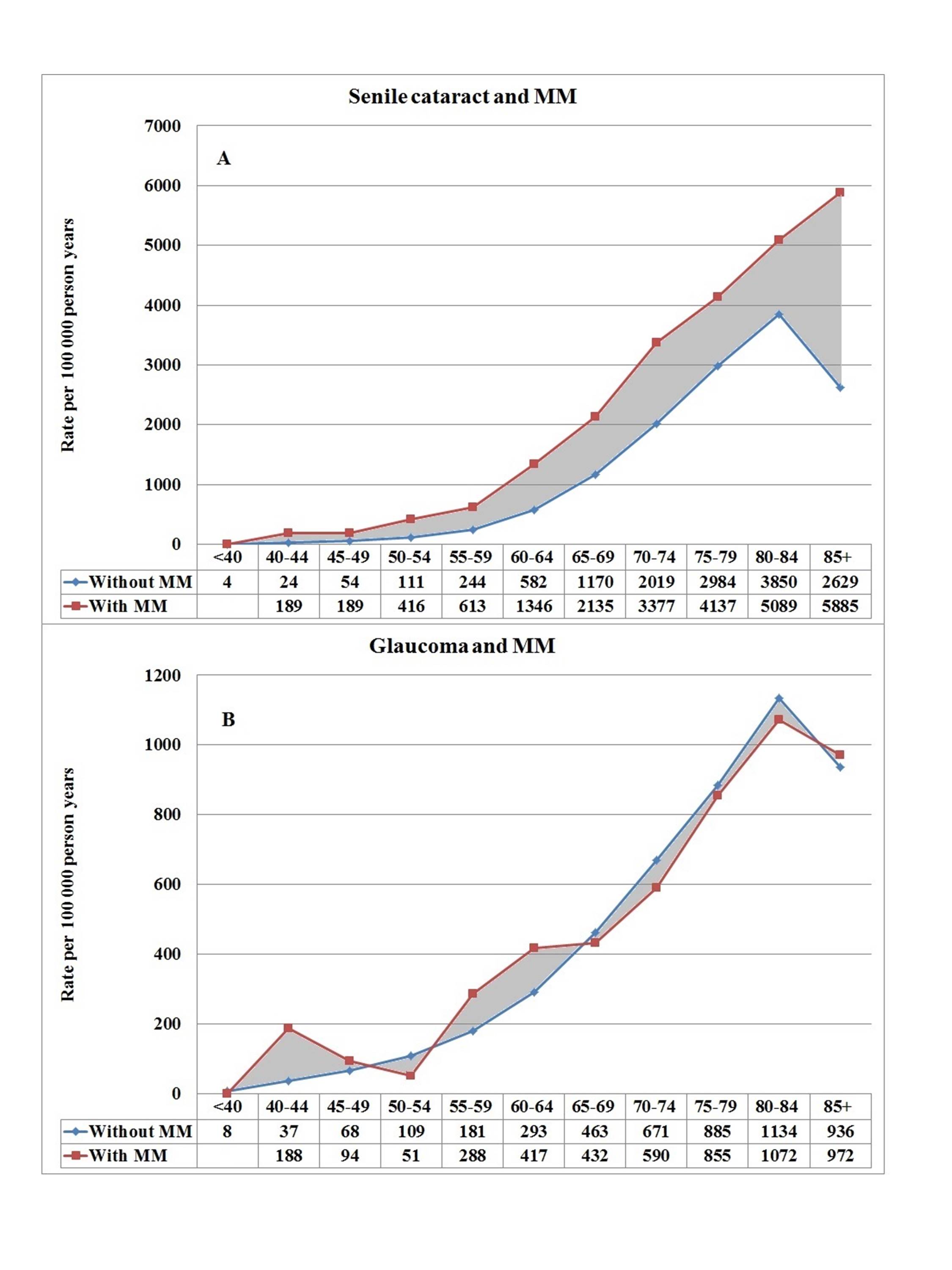


Supplementary Fig 2.


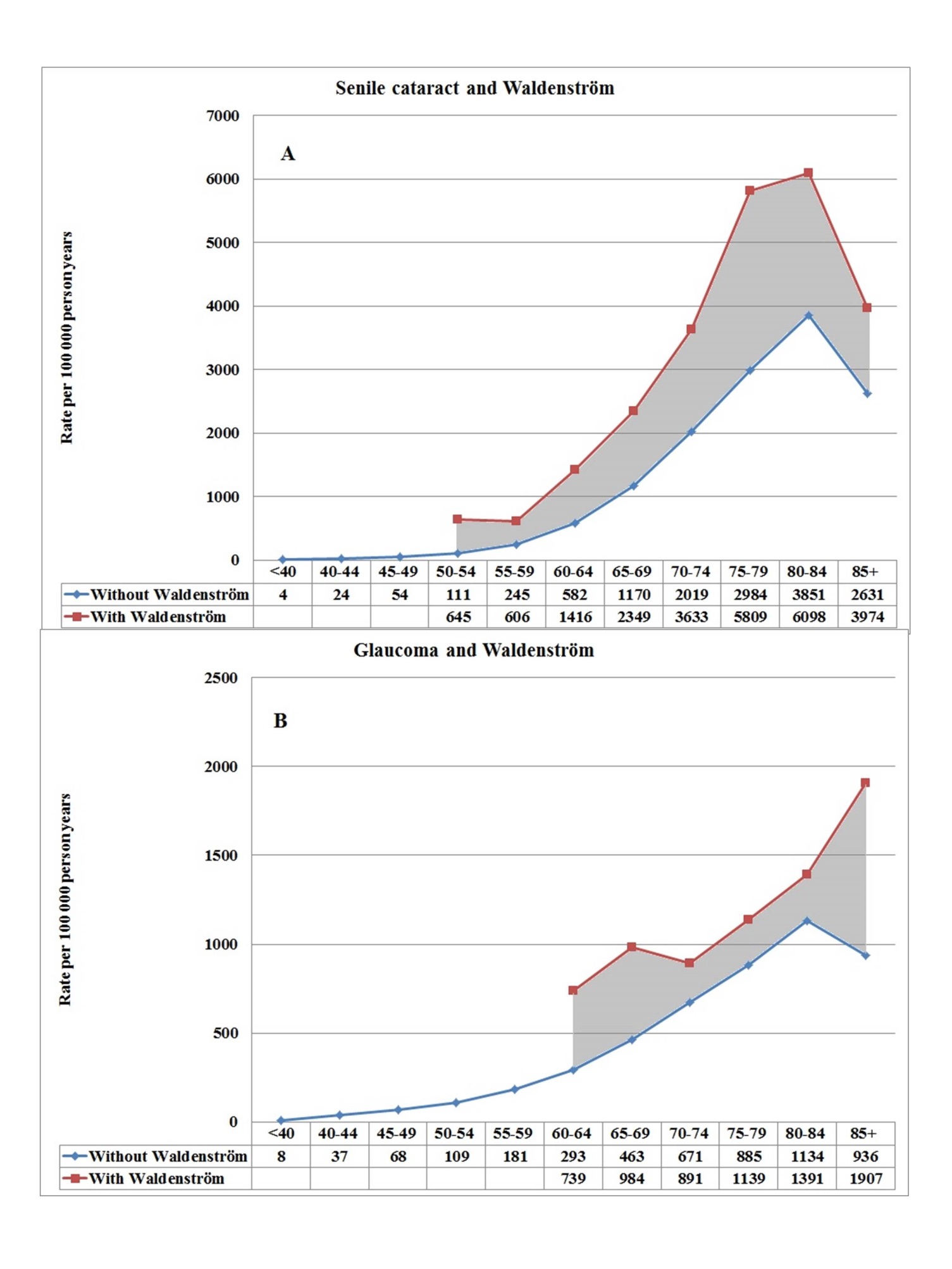


Supplementary Fig 3.


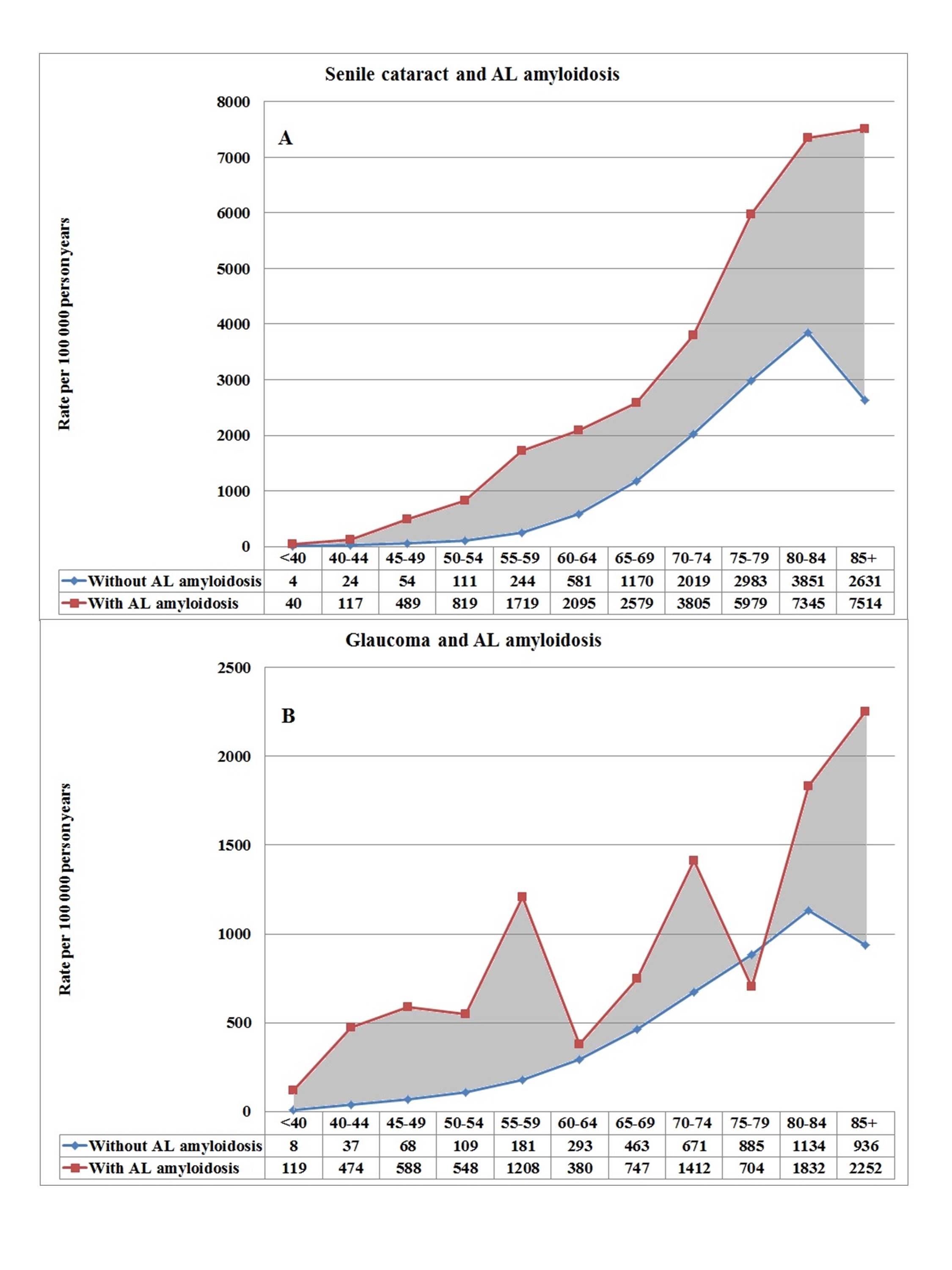

Supplement: Supplementary Information [file srep28500-s1.doc]
